# Supplementary material for: Long-Term Consequences of COVID-19: A 1-Year Analysis
Source: J Clin Med. 2023 Apr 3;12(7):2673. doi: 10.3390/jcm12072673 (PMC10095027; doi:10.3390/jcm12072673)
Supplement: Supplementary file 1 [file jcm-12-02673-s001.zip › jcm-2302762-supplementary.pdf]

Suppl Table S1. Characteristics of COVID-19 patients who received the questionnaire.

	Ambulatory, N = 749[1]	Hospitalized, N = 849[1]	Overall, N = 1,598[1]	p-value[2]
Age	42 (31, 55)	67 (55, 79)	56 (39, 72)	<0.001
Sex				<0.001
Men	39.9% [299]	56.5% [480]	48.7% [779]	
Women	60.1% [450]	43.5% [369]	51.3% [819]	
Cigarette smoking				<0.001
Never-smoker	91.6% [668]	79.9% [678]	85.3% [1,346]	
Former smoker	5.2% [38]	15.7% [133]	10.8% [171]	
Current smoker	3.2% [23]	4.5% [38]	3.9% [61]	
Unknown	20	0	20	
Comorbidities				
Charlson comorbidity index	0.00 (0.00, 1.00)	4.00 (2.00, 6.00)	2.00 (0.00, 5.00)	<0.001
Diabetes	6.3% [47]	24.5% [208]	16.0% [255]	<0.001
Cirrhosis	0.1% [1]	1.9% [16]	1.1% [17]	<0.001
Cancer	3.7% [28]	7.5% [64]	5.8% [92]	0.001
Obesity	7.1% [53]	28.0% [238]	18.2% [291]	<0.001
Overweight and obese	12.4% [93]	56.4% [479]	35.8% [572]	<0.001
Hypertension	15.5% [116]	49.5% [420]	33.5% [536]	<0.001
Atrial fibrillation	0.8% [6]	10.1% [86]	5.8% [92]	<0.001
Coronary disease	2.8% [21]	13.4% [114]	8.4% [135]	<0.001
Stroke	1.3% [10]	7.4% [63]	4.6% [73]	<0.001
Chronic kidney disease	0.8% [6]	13.1% [111]	7.3% [117]	<0.001
COPD	0.7% [5]	6.9% [59]	4.0% [64]	<0.001
Asthma	9.1% [68]	6.4% [54]	7.6% [122]	0.041
Ventilation status				<0.001
Not ventilated	100.0% [749]	90.1% [765]	94.7% [1,514]	
Ventilated	0.0% [0]	9.9% [84]	5.3% [84]	
Discharge				0.5
Alive	100.0% [749]	99.8% [847]	99.9% [1,596]	
Dead	0.0% [0]	0.2% [2]	0.1% [2]	

[1]Median (IQR); % [n] [2]Wilcoxon rank sum test; Pearson's Chi-squared test; Fisher's exact test

Suppl Table S2: Characteristics of COVID-19 ambulatory patients who answered the questionnaire compared to those who did not respond

	Responding Group, N = 252[1]	No responding Group, N = 497[1]	Overall, N = 749[1]	p-value[2]
Age	41 (31, 56)	42 (31, 54)	42 (31, 55)	0.4
Sex				0.047
Men	34.9% [88]	42.5% [211]	39.9% [299]	
Women	65.1% [164]	57.5% [286]	60.1% [450]	
Cigarette smoking				0.4
Never-smoker	93.5% [229]	90.7% [439]	91.6% [668]	
Former smoker	4.1% [10]	5.8% [28]	5.2% [38]	
Current smoker	2.4% [6]	3.5% [17]	3.2% [23]	
Unknown	7	13	20	
Comorbidities				
Charlson comorbidity index	0.00 (0.00, 2.00)	0.00 (0.00, 1.00)	0.00 (0.00, 1.00)	>0.9
Diabetes	4.0% [10]	7.4% [37]	6.3% [47]	0.064
Cirrhosis	0.0% [0]	0.2% [1]	0.1% [1]	>0.9
Cancer	3.6% [9]	3.8% [19]	3.7% [28]	0.9
Obesity	2.8% [7]	9.3% [46]	7.1% [53]	0.001
Overweight and obese	7.1% [18]	15.1% [75]	12.4% [93]	0.002
Hypertension	13.9% [35]	16.3% [81]	15.5% [116]	0.4
Atrial fibrillation	0.4% [1]	1.0% [5]	0.8% [6]	0.7
Coronary disease	2.8% [7]	2.8% [14]	2.8% [21]	>0.9
Stroke	1.6% [4]	1.2% [6]	1.3% [10]	0.7
Chronic kidney disease	0.4% [1]	1.0% [5]	0.8% [6]	0.7
COPD	0.4% [1]	0.8% [4]	0.7% [5]	0.7
Asthma	9.1% [23]	9.1% [45]	9.1% [68]	>0.9
Ventilation status				>0.9
Not ventilated	100.0% [252]	100.0% [497]	100.0% [749]	
Ventilated	0.0% [0]	0.0% [0]	0.0% [0]	

[1]Median (IQR); % [n] [2]Wilcoxon rank sum test; Fisher's exact test; Pearson's Chi-squared test

Suppl Table S3: Characteristics of COVID-19 hospitalized patients who answered the questionnaire compared to those who did not respond

	**Responding Group**, N = 222[1]	**No responding Group**, N = 627[1]	**Overall**, N = 849[1]	**p-value**[2]
**Age**	66 (55, 78)	67 (55, 80)	67 (55, 79)	0.4
**Sex**				0.7
Men	55.4% [123]	56.9% [357]	56.5% [480]	
Women	44.6% [99]	43.1% [270]	43.5% [369]	
**Cigarette smoking**				0.3
Never-smoker	80.2% [178]	79.7% [500]	79.9% [678]	
Former smoker	17.1% [38]	15.2% [95]	15.7% [133]	
Current smoker	2.7% [6]	5.1% [32]	4.5% [38]	
**Comorbidities**				
Charlson comorbidity index	3.00 (2.00, 5.00)	4.00 (2.00, 6.00)	4.00 (2.00, 6.00)	**0.003**
Diabetes	19.4% [43]	26.3% [165]	24.5% [208]	**0.039**
Cirrhosis	0.9% [2]	2.2% [14]	1.9% [16]	0.3
Cancer	6.3% [14]	8.0% [50]	7.5% [64]	0.4
Obesity	30.6% [68]	27.1% [170]	28.0% [238]	0.3
Overweight and obese	60.8% [135]	54.9% [344]	56.4% [479]	0.12
Hypertension	45.9% [102]	50.7% [318]	49.5% [420]	0.2
Atrial fibrillation	9.5% [21]	10.4% [65]	10.1% [86]	0.7
Coronary disease	9.0% [20]	15.0% [94]	13.4% [114]	**0.025**
Stroke	6.3% [14]	7.8% [49]	7.4% [63]	0.5
Chronic kidney disease	8.1% [18]	14.8% [93]	13.1% [111]	**0.011**
COPD	7.7% [17]	6.7% [42]	6.9% [59]	0.6
Asthma	5.4% [12]	6.7% [42]	6.4% [54]	0.5
**Ventilation status**				0.11
Not ventilated	87.4% [194]	91.1% [571]	90.1% [765]	
Ventilated	12.6% [28]	8.9% [56]	9.9% [84]	

[1]Median (IQR); % [n] [2]Wilcoxon rank sum test; Pearson's Chi-squared test; Fisher's exact test

Suppl Table S4: NYHA and CCS scores evaluation comparing ambulatory and hospitalized patients

	Ambulatory, N = 252[1]	Hospitalized, N = 222[1]	Overall, N = 474[1]	p-value[2]
NYHA at COVID				<0.001
1	99.2% [250]	86.5% [192]	93.2% [442]	
2	0.4% [1]	12.6% [28]	6.1% [29]	
3	0.4% [1]	0.9% [2]	0.6% [3]	
NYHA-1 year				<0.001
1	96.4% [243]	84.2% [187]	90.7% [430]	
2	2.4% [6]	13.5% [30]	7.6% [36]	
3	0.8% [2]	1.4% [3]	1.1% [5]	
4	0.4% [1]	0.9% [2]	0.6% [3]	
CCS at COVID				<0.001
0	93.7% [236]	82.4% [183]	88.4% [419]	
1	6.0% [15]	14.4% [32]	9.9% [47]	
2	0.0% [0]	2.7% [6]	1.3% [6]	
3	0.0% [0]	0.0% [0]	0.0% [0]	
4	0.4% [1]	0.5% [1]	0.4% [2]	
CCS-1 year				0.068
0	84.5% [213]	76.1% [169]	80.6% [382]	
1	13.1% [33]	18.9% [42]	15.8% [75]	
2	1.6% [4]	3.6% [8]	2.5% [12]	
3	0.0% [0]	0.9% [2]	0.4% [2]	
4	0.8% [2]	0.5% [1]	0.6% [3]	

[1]% [n] [2]Fisher's exact test

Suppl Table S5: Multivariate analysis of NYHA worsening on the total population

	OR[1]	95% CI[2]	p-value
Age	1.03	1.01, 1.05	0.015
Sex			
• Men	1.00		
• Women	2.31	0.96, 6.04	0.07
Ventilatory status			
• Not ventilated	1.00		
• Ventilated	3.72	0.99, 11.4	0.03

[1]OR: Odds Ratio, [2]CI: confidence interval

Suppl Table S6 : Complete list of 25 neurological symptoms assessed at COVID occurrence

	Ambulatory, N = 252[1]	Hospitalized, N = 222[1]	Overall, N = 474[1]	p-value[2]
Fatigue	90.8% [229]	83.7% [186]	87.5% [415]	0.3
Anosmia	75.8% [191]	43.6% [97]	60.7% [288]	<0.001
Dysgeusia	70.2% [177]	41.8% [93]	56.9% [270]	<0.001
Headaches	76.9% [194]	59.0% [131]	68.5% [325]	<0.001
Feeling slowed down	71.0% [179]	72.0% [160]	71.5% [339]	0.4
Sleepiness	56.7% [143]	60.3% [134]	58.4% [277]	>0.9
"Pressure in the head"	57.1% [144]	41.4% [92]	49.7% [236]	0.032
"Feeling as if in a fog"	46.0% [116]	54.9% [122]	50.2% [238]	0.2
Difficulty focusing	61.1% [154]	59.4% [132]	60.3% [286]	>0.9
"I don't feel like myself"	37.7% [95]	50.0% [111]	43.4% [206]	0.2
"Head spinning"	48.4% [122]	51.3% [114]	49.7% [236]	>0.9
Dizziness	36.1% [91]	45.5% [101]	40.5% [192]	0.4
Neck pain	41.2% [104]	29.2% [65]	35.6% [169]	0.011
Nausea or vomiting	28.5% [72]	30.6% [68]	29.5% [140]	0.5
Confusion	22.2% [56]	39.6% [88]	30.3% [144]	0.001
Problems with balance	29.7% [75]	51.8% [115]	40.1% [190]	<0.001
Photophobia	31.7% [80]	34.6% [77]	33.1% [157]	0.7
Increased sensitivity to noise	25.8% [65]	37.4% [83]	31.2% [148]	0.07
Difficulty moving one or more legs	20.2% [51]	33.3% [74]	26.3% [125]	0.02
Memory problems	32.5% [82]	53.6% [119]	42.4% [201]	<0.001
Difficulty moving one or both arms	19.0% [48]	23.4% [52]	21.1% [100]	0.4
Insomnia	27.3% [69]	47.7% [106]	36.9% [175]	<0.001
Blurred vision	21.8% [55]	33.7% [75]	27.4% [130]	0.002
Tingling, burning sensation	19.4% [49]	29.2% [65]	24.0% [114]	
Decreased sensitivity	15.4% [39]	26.5% [59]	20.6% [98]	

[1]% [n] [2]Pearson's Chi-squared test

Suppl Table S7: Neurological variables significantly different between COVID occurrence and 1-year follow up based on the age category

	**18-49**, N = 194[1]	**50-64**, N = 134[1]	**65-79**, N = 95[1]	**> 80**, N = 51[1]	**Overall**, N = 474[1]	**p-value**[2]
Headaches	-2.00 (-4.00, -1.00)	-2.00 (-4.00, 0.00)	0.00 (-2.00, 0.00)	0.00 (-1.00, 0.00)	-1.00 (-3.00, 0.00)	<0.001
Anosmia	-4.00 (-6.00, -1.00)	-1.00 (-4.00, 0.00)	0.00 (-2.00, 0.00)	0.00 (-1.00, 0.00)	-1.00 (-4.00, 0.00)	<0.001
Dysgeusia	-4.00 (-6.00, 0.00)	-1.00 (-4.00, 0.00)	0.00 (-2.00, 0.00)	0.00 (-0.75, 0.00)	-1.00 (-4.25, 0.00)	<0.001
Feeling slowed down	-2.00 (-4.00, 0.00)	-2.00 (-4.00, 0.00)	-1.00 (-2.00, 0.00)	0.00 (-1.00, 0.00)	-1.00 (-3.00, 0.00)	<0.001
Difficulty focusing	-1.00 (-3.00, 0.00)	-1.00 (-3.00, 0.00)	0.00 (-2.00, 0.00)	0.00 (-1.00, 0.00)	0.00 (-3.00, 0.00)	0.2
Fatigue	-3.00 (-4.25, -1.00)	-2.00 (-4.00, 0.00)	-1.00 (-4.00, 0.00)	-1.00 (-2.00, 0.00)	-2.00 (-4.00, 0.00)	<0.001
Sleepiness	-1.00 (-3.50, 0.00)	-1.00 (-3.00, 0.00)	-1.00 (-3.00, 0.00)	0.00 (-1.00, 0.00)	-1.00 (-3.00, 0.00)	0.014

Suppl Table S8: Principal component analysis of the neurological items

Items/ Principal components	"Neurologic symptoms"	"Anosmia/dysgeusia"
Headaches	0.657	
**Pressure in the head**	**0.719**	
Neck pain	0.597	
Nausea or vomiting	0.521	
Head spinning	0.712	
Blurred vision	0.663	
**Anosmia**		**0.869**
**Dysgeusia**		**0.849**
Problems with balance	0.634	
Dizziness	0.712	
Photophobia	0.697	
Increased sensitivity to noise	0.716	
**Feeling slowed down**	**0.815**	
**"Feeeling as if in a fog"**	**0.760**	
"I do not feel like myself"	0.721	
**Difficulty focusing**	**0.766**	
**Memory problems**	**0.749**	
Fatigue	0.726	
Confusion	0.703	
Sleepiness	0.713	
Insomnia	0.607	
% explained variability	44.7%	8%
Cumulative explained variability	44.7%	52.7%

Suppl Table S9: Comparison STAI, CESD, MVEQ, PQSI scores in 18-49 years-old (A) men and (B) women between CoLaus¦PsyCoLaus and SLOCO (global and splitting ambulatory and hospitalized)

(A)

	**PsyCoLaus 18-49 men**		**SLOCO 18-49 men**			**SLOCO 18-49 men hospitalized**			**SLOCO 18-49 men ambulatory**		
	n	Mean+/-SD	n	Mean+/-SD	p*	n	Mean+/-SD	p*	n	Mean+/-SD	p*
**STAI (anxiety)**											
State : total score	61	35+/-11	59	34+/-12	0.87	13	37+/-17	0.5	46	34+/-11	0.59
**CESD (depression)**											
Total score	61	11+/-8	58	10+/-9	0.64	13	11+/-11	0.86	45	10+/-8	0.48
**MVEQ (fatigue)**											
Total score	61	6+/-5	58	6+/-5	0.88	14	5.71	0.83	44	6+/-5	0.78
**PSQI (sleep)**											
Global score	ND	ND	58	5+/-3		14	5.21	44		4+/-3	

(B)

	**PsyCoLaus 18-49 women**		**SLOCO 18-49 women**			**SLOCO 18-49 women hospitalized**			**SLOCO 18-49 women ambulatory**		
	n	Mean+/-SD	n	Mean+/-SD	p*	n	Mean+/-SD	p*	n	Mean+/-SD	p*
**STAI (anxiety)**											
State : total score	133	33+/-11	132	38+/-12	**0.001**	17	38+/-13	0.073	115	38+/-13	**0.003**
**CESD (depression)**											
Total score	133	13+/-9	125	13+/-10	0.73	18	12+/-9	0.87	107	13+/-10	0.63
**MVEQ (fatigue)**											
Total score	133	7+/-5	129	8+/-6		16	9+/-6	0.21	113	8+/-6	0.063
**PSQI (sleep)**											
Global score	ND	ND	131	6+/-4		17	7+/-4		14	5+/-4	

STAI : State and Trait Anxiety, CESD : Center for Epidemiologic Studies- Depression score, MVEQ : Maastricht Vital Exhaustion Questionnaire, PSQI : Pittsburgh Sleep Quality Index score.

Suppl Table S10: Comparison STAI, CESD, MVEQ, PQSI scores in $\geq$50 years-old (A) men and (B) women between CoLaus¦PsyCoLaus and SLOCO (global and splitting ambulatory and hospitalized)

(A)

	**PsyCoLaus >50 men**		**SLOCO >50 men**			**SLOCO >50 men hospitalized**			**SLOCO >50 men ambulatory**		
	n	Mean+/-SD	n	Mean+/-SD	p*	n	Mean+/-SD	p*	n	Mean+/-SD	p*
**STAI (anxiety)**											
State : total score	2173	30+/-10	146	34+/-12	**<0.01**	108	34+/-13	**0.0002**	38	34.6	**0.01**
**CESD (depression)**											
Total score	2173	10+/-8	144	12+/-10	**<0.01**	104	13+/-11	**0.0002**	40	10+/-8	0.75
**MVEQ (fatigue)**											
Total score	1068	4+/-4	147	8+/-5	**<0.001**	108	8+/-5	**<0.001**	39	7+/-6	**0.0006**
**PSQI (sleep)**											
Global score	1932	5+/-3	140	6+/-4		102	6+/-4	**0.0008**	38	5+/-3	0.08

(B)

	**PsyCoLaus >50 women**		**SLOCO >50 women**			**SLOCO >50 women hospitalized**			**SLOCO >50 women ambulatory**		
	n	Mean+/-SD	n	Mean+/-SD	p*	n	Mean+/-SD	p*	n	Mean+/-SD	p*
**STAI (anxiety)**											
State : total score	1504	32+/-10	123	36+/-14	**<0.001**	76	37+/-14	**0.0001**	47	36+/-13	**0.009**
**CESD (depression)**											
Total score	2543	11+/-9	119	14+/-10	**0.003**	76	15+/-10	**0.0002**	43	11+/-9	0.97
**MVEQ (fatigue)**											
Total score	1371	6+/-5	124	10+/-6	**<0.001**	77	10+/-6	**<0.001**	47	9+/-6	**<0.001**
**PSQI (sleep)**											
Global score	2179	5+/-5	126	7+/-4	**0.0005**	79	8+/-4	**0.00007**	47	6+/-4	0.61

STAI : State and Trait Anxiety, CESD : Center for Epidemiologic Studies- Depression score, MVEQ : Maastricht Vital Exhaustion Questionnaire, PSQI : Pittsburgh Sleep Quality Index score.

Suppl Table S11: Discrimination between clusters for the variables used for the partition around medoids. Data are shown as means ± standard deviations, and compared using the Student test.

**12-month assessment after Covid infection**	**"Long Covid" cluster** (N = 124)	**"Others" cluster** (N = 350)	**P-value**
Neurologic symptoms			
Pressure in the head	2.45 ± 1.74	1.15 ± 0.58	<0.001
Feeling slowed down	3.99 ± 1.78	1.4 ± 0.82	<0.001
Feeling as if in a fog	2.61 ± 1.71	1.1 ± 0.4	<0.001
Difficulty focusing	3.87 ± 1.78	1.4 ± 0.85	<0.001
Memory problems	3.89 ± 1.83	1.48 ± 0.93	<0.001
Anosmia/dysgeusia			
Anosmia	2.01 ± 1.62	1.51 ± 1.29	<0.001
Dysgueusia	1.83 ± 1.48	1.35 ± 0.98	<0.001
NYHA score	1.23 ± 0.57	1.07 ± 0.31	<0.001
CCS score	0.56 ± 0.83	0.13 ± 0.41	<0.001
CSD score	20.23 ± 10.56	9.78 ± 7.46	<0.001
CSD somatic	5.87 ± 5.02	2.01 ± 2.72	<0.001
CSD depression	5.86 ± 3.2	3.67 ± 3.17	<0.001
CSD relation	8.31 ± 3.74	3.72 ± 3.04	<0.001
CSD positive affection	0.93 ± 1.41	0.35 ± 0.75	<0.001
STAI score	46.2 ± 14.12	32.27 ± 10.15	<0.001
Maastricht score	13.6 ± 3.2	6.18 ± 4.79	<0.001
PSQI score	8.43 ± 4.13	4.89 ± 3.05	<0.001

Suppl Table S12: Baseline patient characteristics across clusters. Data are shown as means ± standard deviations or as numbers and percentages, and were compared across clusters using the Student test, the χ2 test or the Fisher test, as appropriate.

**Baseline characteristics**	**"Long Covid" cluster** (N = 124)	**"Others" cluster** (N = 350)	**P-value**
Age	56.91 ± 17.53	53.38 ± 19.36	0.07
Male	46 (37.1%)	165 (47.1%)	0.07
Body Mass Index*	27.18 ± 4.34	27.44 ± 5.69	0.72
Obesity*	20 (27.8%)	55 (36.7%)	0.99
Overweight*	49 (39.5%)	104 (69.3%)	0.06
Active smoker	5 (4.1%)	7 (2%)	0.45
Prior smoker	12 (9.9%)	36 (10.4%)	
No smoker	104 (85.9%)	303 (87.6%)	
Prior chronic disease	58 (47.1%)	121 (34.8%)	0.02
CCI comorbidity score	1.5 ± 2.5	2.13 ± 2.47	0.15
Hypertension	43 (34.7%)	94 (26.9%)	0.12
Atrial fibrillation	11 (8.9%)	11 (3.1%)	0.02
Coronary heart disease	5 (4%)	22 (6.3%)	0.48
Stroke	9 (7.2%)	9 (2.6%)	0.04
Chronic kidney disease	5 (4%)	14 (4%)	0.99
COPD	7 (5.6%)	11 (3.1%)	0.33
NYHA score	1.12 ± 0.35	1.06 ± 0.26	0.03
CCS score	0.31 ± 0.68	0.08 ± 0.31	<0.001
Mother language			
French	81 (65.3%)	261 (74.6%)	0.01
German	4 (3.2%)	16 (4.6%)	
Italian	3 (2.4%)	18 (5.1%)	
English	1 (0.8%)	5 (1.4%)	
Other	35 (28.2%)	50 (14.3%)	

*only available in the subgroup admitted to hospital during Covid infection: 72 patients in the "Long Covid" cluster and 150 in the "Others" cluster.

Suppl Table S13: Baseline patient characteristics across clusters. Data are shown as means ± standard deviations or as numbers and percentages, and were compared across clusters using the Student test, the χ2 test or the Fisher test, as appropriate.

**Patient characteristics during the Covid infection**	**"Long Covid" cluster** (N = 124)	**"Others" cluster** (N = 350)	**P-value**
Symptoms			
Headaches	4.42 ± 2.24	3.32 ± 2.16	<0.001
"Pressure in the head"	3.93 ± 2.39	2.52 ± 2.05	<0.001
Neck pain	3.08 ± 2.27	1.88 ± 1.56	<0.001
Nausea	2.69 ± 2.1	1.71 ± 1.55	<0.001
"Head spinning"	3.76 ± 2.05	2.29 ± 1.87	<0.001
Blurred vision	2.81 ± 2	1.48 ± 1.26	<0.001
Anosmia	3.8 ± 2.61	3.81 ± 2.6	0.95
Dysgueusia	3.83 ± 2.62	3.52 ± 2.54	0.25
Problems with balance	3.44 ± 2.17	1.85 ± 1.55	<0.001
Dizziness	3.4 ± 2.08	1.93 ± 1.69	<0.001
Trouble moving arms	2.18 ± 1.86	1.48 ± 1.27	<0.001
Trouble moving legs	2.36 ± 1.93	1.64 ± 1.44	<0.001
Decreased sensitivity	2.28 ± 1.64	1.29 ± 0.95	<0.001
Tingling, burning sensation	2.8 ± 1.92	1.4 ± 1.16	<0.001
Photophobia	3.07 ± 2.11	1.64 ± 1.39	<0.001
Increased sensitivity to noise	3.19 ± 2.14	1.55 ± 1.28	<0.001
Feeling slowed down	5.15 ± 1.86	3.31 ± 2.13	<0.001
"Feeling as if in a fog"	4.21 ± 2.1	2.32 ± 1.9	<0.001
"I do not feel like myself"	4.19 ± 2.39	2.2 ± 1.93	<0.001
Difficulty focusing	4.68 ± 2.07	2.7 ± 1.98	<0.001
Memory problems	4.12 ± 2.28	1.96 ± 1.7	<0.001
Fatigue	5.94 ± 1.66	4.9 ± 2.08	<0.001
Confusion	3.45 ± 2.23	1.55 ± 1.37	<0.001
Sleepiness	4.51 ± 2.19	2.91 ± 2.15	<0.001
Insomnia	3.6 ± 2.42	1.88 ± 1.65	<0.001
Admission to hospital	72 (58.1%)	150 (42.8%)	0.005
Mechanical ventilation*	13 (10.5%)	15 (4.3%)	0.02
Symptoms prior to hospital admission*			
Cough	54 (75%)	101 (67.3%)	0.16
Tiredness	49 (68%)	97 (64.7%)	0.59
Dyspnoea	48 (66.7%)	91 (60.7%)	0.42
Myalgia	23 (31.9%)	35 (23.3%)	0.12
Nausea	20 (27.8%)	31 (20.7%)	0.31
Diarrhea	19 (26.4%)	36 (24%)	0.66
Faintness	4 (5.6%)	4 (2.7%)	0.49
Confusion	3 (4.2%)	2 (1.3%)	0.40
Number of days with symptoms prior to hospital admission*	4.77 ± 5.12	3.59 ± 5.47	0.97

* in the subgroup admitted to hospital during Covid infection: 72 patients in the "Long Covid "cluster and 150 in the "Others" cluster.
